# Diabetesmanagement im Krankenhaus (Update 2023)

**DOI:** 10.1007/s00508-023-02177-5

**Published:** 2023-04-20

**Authors:** Julia K. Mader, Johanna M. Brix, Felix Aberer, Alexander Vonbank, Michael Resl, Daniel A. Hochfellner, Claudia Ress, Thomas R. Pieber, Lars Stechemesser, Harald Sourij

**Affiliations:** 1 Klinische Abteilung für Endokrinologie und Diabetologie, Universitätsklinik für Innere Medizin, Medizinische Universität Graz, Auenbruggerplatz 15, 8036 Graz, Österreich; 2 Medizinische Abteilung mit Diabetologie, Endokrinologie und Nephrologie, Klinik Landstraße, Wien, Österreich; 3 Innere Medizin I mit Kardiologie, Angiologie, Endokrinologie, Diabetologie und Intensivmedizin, Akademisches Lehrkrankenhaus Feldkirch, Feldkirch, Österreich; 4 Abteilung für Innere Medizin, Konventhospital der Barmherzigen Brüder Linz, Linz, Österreich; 5 Innere Medizin, Department I, Medizinische Universität Innsbruck, Innsbruck, Österreich; 6 Universitätsklinik für Innere Medizin I, Paracelsus Medizinische Privatuniversität – Landeskrankenhaus, Salzburg, Österreich

**Keywords:** Krankenhausdiabetesmanagement, Insulintherapie, Orale Antidiabetika, Erwachsene mit Diabetes mellitus, Normalstation, Hospital diabetes management, Insulin, Therapy, Oral antihyperglycemic drugs, Adults with diabetes mellitus, General ward

## Abstract

Dieses Positionspapier beinhaltet die Empfehlungen der Österreichischen Diabetes Gesellschaft zum Management von erwachsenen Patient:innen mit Diabetes mellitus während stationärer Aufenthalte und basiert auf aktueller Evidenz zu Blutglukosezielbereichen, Insulintherapie und Therapie mit oralen/injizierbaren Antidiabetika während stationärer Aufenthalte. Zusätzlich werden Spezialsituationen wie intravenöse Insulintherapie, begleitende Steroidtherapie sowie die Anwendung von Diabetestechnologie im stationären Bereich diskutiert.

## Abstract (en)

This position statement presents the recommendations of the Austrian Diabetes Association for diabetes management of adult patients during inpatient stay. It is based on the current evidence with respect to blood glucose targets, insulin therapy and treatment with oral/injectable antidiabetic drugs during inpatient hospitalization. Additionally, special circumstances such as intravenous insulin therapy, concomitant therapy with glucocorticoids and use of diabetes technology during hospitalization are discussed.

## Prävalenz von Hyperglykämien im Krankenhaus

Epidemiologische Daten zeigen, dass – vereinbart mit der globalen Zunahme an Diabeteserkrankungen – auch die Anzahl an Patient:innen mit Diabetes und Hyperglykämien im Krankenhaus deutlich ansteigt, wobei die amerikanische Diabetesgesellschaft (ADA) eine Nüchternblutglukose über 140 mg/dl als Hyperglykämie definiert.

Die sogenannte Stresshyperglykämie beschreibt den Zustand erhöhter Blutglukosewerte bei akuten Erkrankungen und tritt als Folge von meist kurzfristigen metabolischen, inflammatorischen und hormonellen Dysregulationen auf. Die Stresshyperglykämie stellt meist ein reversibles Begleitphänomen einer akuten Erkrankung dar. Allerdings persistiert die Hyperglykämie häufig, wenn sie eine Demaskierung einer vorbestehenden Glukosetoleranzstörung darstellt. Unabhängig davon zeigte sich, dass die Stresshyperglykämie in verschiedensten Populationen einen potenteren Risikofaktor für gesundheitliche Komplikationen im Krankenhaus darstellt als die Hyperglykämie bei Patient:innen mit vorbekannter Diabeteserkrankung [1].

Unabhängig vom Vorliegen eines vorbekannten Diabetes mellitus wird die weltweite Prävalenz des Auftretens von Hyperglykämien bei hospitalisierten Patient:innen auf 20–40 % geschätzt [2, 3], wobei kritisch kranke Patient:innen auf Intensivstationen in bis zu 70 % Hyperglykämien aufweisen. Die Prävalenz hyperglykämischer Episoden während eines Krankenhausaufenthalts korreliert, wie auch die Diabetesprävalenz, stark mit dem Alter des Patient:innen. So zeigte sich, dass über 75-Jährige mit einer 2,4-fach höheren Wahrscheinlichkeit mit einer Diabetesdiagnose aus dem Krankenhaus entlassen werden als eine Kontrollgruppe unter 65 Lebensjahren [4, 5].

## Auswirkungen von Hyperglykämien im Krankenhaus

Hyperglykämien bei Patient:innen im Krankenhaus stellen sowohl auf Normalstationen als auch auf Intensivstationen einen erheblichen und unabhängigen Risikofaktor für eine erhöhte Mortalität und gesundheitliche Komplikationen wie Infektionen (z. B. Pneumonien) [6] oder Operationskomplikationen [7] dar. Diese Assoziation korreliert einerseits mit der Höhe der Hyperglykämie bei Krankenhausaufnahme und andererseits mit der mittleren Glukose während des gesamten Krankenhausaufenthaltes [8–10].

Abgesehen von der Tatsache, dass hospitalisierte Patient:innen mit Hyperglykämien höhere Krankenhauskosten verursachen [11, 12], besteht auch ein höheres Risiko für längere Krankenhausaufenthalte [13, 14]. Zudem zeigte sich, dass Patient:innen mit Hyperglykämien während des stationären Aufenthalts häufiger eine poststationäre Rehabilitation und/oder einen Transfer in eine medizinisch betreute Wohneinrichtung (z. B. Pflegeheim) in Anspruch nehmen müssen [15].

Neben den ungünstigen Auswirkungen von Hyperglykämien im Krankenhaus selbst benötigen Menschen mit Diabetes mellitus aufgrund der mit dem Diabetes einhergehenden mikro- und makrovaskulären und neuropathischen Spätkomplikationen häufiger akute und geplante stationäre Aufnahmen [16, 17]. Darüber hinaus führen akute diabetische Komplikationen wie das hyperglykämische Koma, die Ketoazidose und iatrogene Hypoglykämie häufig zur Indikationsstellung einer Krankenhauseinweisung [18].

## Blutglukosezielwerte und Blutglukosemessfrequenz bei hospitalisierten Patient:innen

### Kritisch kranke Patient:innen

- Bei anhaltender Hyperglykämie > 180 mg/dl besteht die Indikation für eine Insulintherapie.
- Ein Blutglukosezielbereich von 140–180 mg/dl ist für die meisten Patient:innen anzustreben.
- Ausgewählte Patient:innen können von einer strengeren glykämischen Kontrolle mit einer Blutglukose von 110–140 mg/dl profitieren, wenn diese ohne signifikante Hypoglykämien erreicht werden kann.
- Für die Entscheidung über mögliche Modifizierungen der antidiabetischen Therapie während eines Krankenhausaufenthaltes sind standardisierte Blutglukosezielwerte notwendig.
- Die Blutzuckerzielwerte sind individuell je nach Komorbiditäten, Begleitmedikation, Ernährungsstatus und Aufnahmegrund festzulegen. Durch eine strikte Blutzuckerkontrolle (Blutglukoseziel: 80–110 mg/dl vs. 180–200 mg/dl) konnte in der Leuven-Studie auf einer chirurgischen Intensivstation eine Reduktion der Mortalität erreicht werden. Ein ähnlicher Ansatz führte jedoch in der NICE-SUGAR-Studie sogar zu einer höheren Mortalität in der Patient:innengruppe mit niedrigeren Blutzuckerzielwerten. Heterogene Patient:innenkollektive und Therapieschemata sind diesbezüglich nach wie vor für inkonklusive Empfehlungen verantwortlich. Eine Metaanalyse zeigte beispielsweise eine erhöhte Mortalität bei hospitalisierten Patient:innen, bei welchen die Blutzuckereinstellung zu strikt gewählt wurde [19].

### Nicht kritisch kranke Patient:innen

- Die Evidenz für einen eng definierten Blutzuckerzielbereich nicht kritisch kranker Patient:innen ist nur eingeschränkt verfügbar, daher musste man sich bei der Definition von Zielbereichen für die Normalstation an die Empfehlungen aus dem intensivmedizinischen Bereich anlehnen.
- Ab einer Blutglukose > 140 mg/dl sollte eine Evaluierung von Ernährung und antidiabetischer Medikation erfolgen.
- Bei persistierender Blutglukose > 180 mg/dl besteht bei hospitalisierten Patient:innen die Indikation für eine Insulintherapie. Unter laufender Insulintherapie wird für den Großteil nicht kritisch kranker Patient:innen ein Blutglukosezielbereich von 140–180 mg/dl angestrebt.
- Bei strengeren Blutglukosezielbereichen (110–140 mg/dl) ist auf eine Vermeidung von signifikanten Hypoglykämien zu achten [20].
- Hypoglykämien 70 mg/dl sollten unter stationären Bedingungen detektiert und dokumentiert werden, und etwaige Therapieadaptierungen sind durchzuführen. [21].
- Bei terminal kranken Patient:innen mit schweren Begleiterkrankungen kann ein individuell höherer Blutglukosezielbereich festgelegt werden.
- Für die Erreichung der Therapieziele im Krankenhaus sind im Vergleich zur Therapieevaluierung zu Hause meist engmaschigere Blutglukosekontrollen notwendig. Bei guter und stabiler Blutglukoseeinstellung auch unter stationären Verhältnissen können die Empfehlungen aus dem Kapitel „Blutglukoseselbstkontrolle“ herangezogen werden. Eine Kontrolle der Blutglukose vor den Mahlzeiten sollte erfolgen. Wenn Patient:innen nicht essen, ist eine Blutglukosemessung zumindest alle 4–6 h durchzuführen [22]. Bei ausgeprägten Hyperglykämien, Hypoglykämien oder hoher glykämischer Variabilität ist meist zumindest ein 7 –Punkt-Profil indiziert. Eine intravenöse Insulintherapie ist alle 30–120 min mittels Blutglukosemessung zu evaluieren.

## Insulintherapie bei hospitalisierten Patient:innen

Ein großer Teil der Krankenhausaufenthalte von Patient:innen mit Diabetes mellitus erfolgt nicht wegen der Diabeteseinstellung per se, sondern aufgrund von Komorbiditäten. Eine Folge davon ist, dass während des stationären Aufenthaltes nicht genügend auf die Diabeteseinstellung geachtet wird, speziell wenn sich Patient:innen nicht auf internistischen Abteilungen befinden. Eine dauerhafte Hyperglykämie ist mit einem schlechteren Outcome bei stationären Patient:innen assoziiert [23].

Die Art der Diabeteserkrankung (Diabetes mellitus Typ 1, Diabetes mellitus Typ 2, „Maturity Onset Diabetes of the Young“ [MODY] etc.) sollte aus der Krankenakte klar ersichtlich sein, nicht zuletzt auch damit gravierende Fehler, wie z. B. das vollständige Absetzen/Pausieren einer Insulintherapie bei Patient:innen mit Diabetes mellitus Typ 1, vermieden werden können. Ein aktueller HbA_1c_-Wert sollte vorliegen bzw. erhoben werden, da der HbA_1c_-Wert auch der Unterscheidung dient, ob eine längerfristige hyperglykämische Situation besteht oder die Blutglukoseerhöhung auf eine akute Blutzuckererhöhung zurückzuführen ist. Bei der HbA_1c_-Bestimmung muss berücksichtig werden, dass durch Anämien, Erythrozytenkonzentratgabe, schwere Nieren- oder Lebererkrankungen der Wert verfälscht sein kann [24].

Ein aktives Diabetesmanagement unter Einbeziehung der Fähigkeiten des Selbstmanagements des Patient:innen wird dringend empfohlen. Bei Patient:innen mit nicht lebensbedrohlichen Erkrankungen sollte die Diabeteseinstellung entsprechend den individuell vereinbarten Therapiezielen erfolgen.

Eine Insulintherapie ist aufgrund der Wirksamkeit, der Steuerbarkeit und der fehlenden Medikamenteninteraktionen der beste Weg, eine Hyperglykämie bei hospitalisierten Patient:innen, im speziellen bei kritisch kranken Patient:innen, zu behandeln, und ist daher das Mittel der Wahl.

## Subkutane Insulintherapie

Die subkutane Insulintherapie ist der bevorzugte Weg der Blutglukosesenkung bei nicht kritisch kranken hospitalisierten Patient:innen außerhalb von Überwachungs- und Intensivstationen. Dabei ist eine basalorientierte Insulintherapie mit zusätzlicher Gabe von Bolusinsulin bei Patient:innen mit regelmäßiger Nahrungsaufnahme zu bevorzugen [25, 26]. Obwohl eine Mischinsulintherapie mit zweimal täglicher Gabe ebenfalls verwendet werden kann, zeigte sich in Studien, dass es dabei zu einem höheren Hypoglykämierisiko kommt [27].

**Figure 1:**
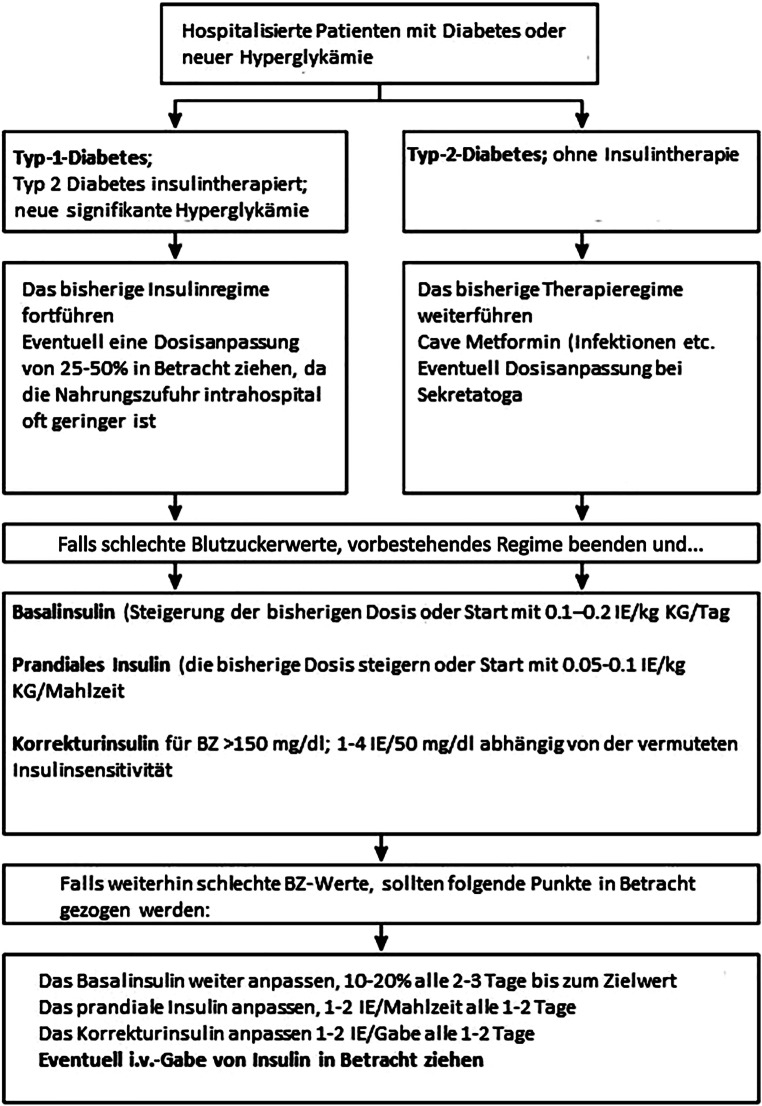

Vor jeder Mahlzeit sollte eine Blutglukosemessung erfolgen. Der Insulintagesbedarf beginnt für die meisten Patient:innen bei 0,3–0,5 IE/kg Körpergewicht [28, 29]. Startdosen über 0,6–0,8 IE/kg Körpergewicht sind mit einem bis zu dreifach erhöhten Hypoglykämierisiko verbunden. Bei älteren Patient:innen (> 70 Jahre) und Patient:innen mit eingeschränkter Nierenfunktion verringert eine angepasste Dosis von 0,2–0,3 IE/kg Körpergewicht das Hypoglykämierisiko [30]. Sollten Patient:innen nüchtern bleiben müssen (z. B. vor einer Operation) oder nehmen Patient:innen nur sehr kleine Mahlzeiten zu sich, ist es möglich nur Korrekturinsulin zu verabreichen. Zu berücksichtigen ist, dass auch eine stabile Basalinsulintherapie präoperativ nicht abgesetzt oder pausiert werden sollte [31]. Ein möglicher Algorithmus wird in Abb. 1 dargestellt. Computergesteuerte, automatisierte Systeme haben durch einen computergestützten Algorithmus zu einer schnelleren Verbesserung der Insulintherapie geführt und zeigten bereits erste überzeugende Daten [32, 33].

## Management bei Insulintherapie im Krankenhaus

Die Durchführung unterschiedlicher Insulintherapieformen stellt v. a. auch für das Pflegepersonal eine große Herausforderung dar. Bei vielen Abteilungen liegt die Behandlung der Blutglukosewerte nicht im Fokus, was häufig Schwierigkeiten in der korrekten Therapieführung bedingt. Die folgende pragmatische Anleitung soll als Anhaltspunkt für die Kooperation zwischen den medizinischen Berufsgruppen dienen und Fehlanwendungen der Therapie verhindern.

1. Grundregeln der Insulintherapie
  Lang wirksame (Basal‑)Insuline (NPH-Insulin, Insulin detemir, Insulin glargin, Insulin glargin U300, Insulin degludec) zum verordneten Zeitpunkt verabreichen. Diese Insuline werden typischerweise täglich in derselben Dosierung verabreicht.
  Kurz wirksame oder ultrakurz wirksame (prandiale) Insuline (Humaninsulin, Insulin aspart, Insulin glulisin, Insulin lispro, faster Insulin aspart, ultra-rapid Insulin lispro) immer unmittelbar vor der Mahlzeit applizieren. Diese werden nach aktuellem Blutglukosewert und Kohlenhydratgehalt der geplanten Mahlzeit dosiert.
  Mischinsuline (z. B. biphasisches Insulin aspart/Insulin aspart Protamin, biphasisches Insulin lispro/Insulin lispro Protamin, biphasisches Humaninsulin/Protamininsulin, Insulin degludec/Insulin aspart; in verschiedenen Mischverhältnissen) immer unmittelbar vor der Mahlzeit des Verordnungszeitpunktes applizieren.
  Bei Hypoglykämien (Blutglukosewerte < 70 mg/dl) vor der Insulingabe rasche Korrektur der Hypoglykämie mit rasch wirksamen Kohlehydraten und anschließend engmaschige Nachmessung der Blutglukose (cave: protrahierte Hypoglykämien). Bei Normalisierung Verabreichung der für die nun folgende Mahlzeit festgesetzten Insulindosis.
2. Blutglukosemessungen – Wie oft und bei wem?
  In den ersten Tagen nach der Aufnahme bei bestehendem Diabetes mellitus, bei Neudiagnose eines Diabetes mellitus, Neueinstellung auf eine Insulintherapie oder Wechsel des Therapieschemas mindestens dreimal täglich vor den Mahlzeiten sowie vor dem Zubettgehen
  15 min nach einer Hypoglykämie und getroffenen Gegenmaßnahmen
  Bei stabilen Blutglukosewerten und einer Therapie mit oralen/injizierbaren Antidiabetika kann eine Reduktion der Messfrequenz auf ein- bis zweimal täglich nach einigen Tagen in Erwägung gezogen werden
  Bei Mischinsulintherapie – je nach Anordnung – auch 2 h nach der Mahlzeit
  Bei intensivierter Insulintherapie (Basalinsulin + kurz wirksames Insulin) Messungen optimalerweise vor den Mahlzeiten und 2 h danach
  Reduktion der Messfrequenz je nach Verlauf der Blutglukosewerte

## Intravenöse Insulintherapie

Kritisch kranke Patient:innen auf Intensiv- und Überwachungsstationen, aber auch Patient:innen mit diabetischer Ketoazidose und/oder hyperglykämischen, hyperosmolaren Entgleisungen sollten primär mittels einer kontinuierlichen intravenösen Insulingabe behandelt werden [34, 35]. Klare Vorteile der intravenösen Insulingabe sind die bessere Steuerbarkeit, die raschere Möglichkeit, auf Entgleisungen zu reagieren, und die bessere kinetische Insulinwirkung mit einer kurzen Halbwertszeit durch eine intravenöse Applikation. In Österreich sind Human- und 4 Analoginsuline mit schnellem Wirkeintritt für die intravenöse Verabreichung verfügbar (Humaninsulin, Insulin aspart, Insulin glulisin, Insulin lispro, Insulin faster lispro).

Die Verabreichung sollte mittels Perfusor mit 50 IE kurz wirksamen Insulinanaloga oder Humaninsulin in 50 ml NaCl 0,9 % erfolgen. Zahlreiche intravenöse Infusionsprotokolle zeigten sich als effektiv beim Erreichen der Zielwerte mit einem niedrigen Hypoglykämierisiko [32, 36–38]. Ein mögliches Protokoll wird in Tab. 1 dargestellt [39]. Sollte eine orale Nahrungszufuhr erfolgen, ist 1 Broteinheit ([BE] 12 g Kohlenhydrate) mit einer Insulineinheit als i.v.-Bolus abzudecken. Sollte der Blutglukosespiegel nach 1 h um weniger als 10 % vom Ausgangswert fallen, kann die Insulinmenge auf bis zu 0,15–0,20 IE/kg Körpergewicht pro Stunde gesteigert werden [32, 40].

**Table 1:** 

Blutglukosewert (mg/dl)	Insulindosis (ml/h = IE/h)
< 80	Perfusorpause und Kontrolle in 30 min
81–120	0,7
121–150	1,0
151–180	1,5
181–210	2,0
211–240	2,5
241–270	3,0
271–300	3,5
301–330	4,0
331–360	4,5
361–390	5,0
391–420	5,5
421–450	6,0

Bei Patient:innen ohne Ketoazidose und kontinuierlicher Insulininfusionstherapie ist eine Umstellung zu empfehlen, wenn eine Besserung des Gesundheitszustandes (Extubation, Aufnahme der enteralen Ernährung etc.) erreicht ist und die Höhe der Blutglukosewerte eine Beendigung der intravenösen Insulingabe erlaubt. Bei Patient:innen mit einer Ketoazidose kann eine Umstellung erfolgen, wenn der pH sich normalisiert hat und die Blutglukosewerte zufriedenstellend sind. Weiters müssen bei einer DKA die Elektrolyte (insbesondere Kalium und Natrium) engmaschig kontrolliert und gegebenenfalls substituiert werden. Nähere Details sind in der Guideline zu diabetischer Ketoazidose zu finden.

Eine Umstellung der intravenösen Insulingabe auf eine subkutane Gabe sollte überlappend erfolgen. Nach der ersten subkutanen Gabe eines Basalinsulins sollte der Insulinperfusor noch für 2 h weitergeführt werden.

Als subkutane Startdosis für das Basalinsulin werden 60 % der letzten, kumulativen intravenösen Tagesdosis (über die letzten 24 h) verwendet, wobei eine eventuell durchgeführte Umstellung der Ernährung (parenteral auf enteral) unbedingt bei der Abschätzung des Insulinbedarfs berücksichtigt werden muss. Bei Beginn der enteralen Ernährung sind die anderen 40 % regelmäßig auf die Hauptmahlzeiten aufzuteilen [34, 41].

## Prä- bzw. intraoperatives Management

Für die prä- bzw. intraoperative Phase wird folgendes Vorgehen empfohlen [42, 43].

### Kurze Eingriffe

Subkutane Insulingaben können beibehalten werden, wenn durch die Operation nicht mehr als 1 bis 2 Mahlzeiten versäumt werden.

#### Kleine Eingriffe am Morgen, durch die das Frühstück nur verzögert wird

Insulingabe verschieben, erst vor dem Frühstück applizieren.

Bei 1 -mal täglicher Gabe eines lang wirksamen Insulins: keine Änderung erforderlich, wenn die Dosis präoperativ adäquat war. Bei eher niedrigen präoperativen Blutglukosewerten Dosisreduktion um 20 % erwägen.

#### Wenn Frühstück und Mittagessen ausfallen

Kein kurz wirksames Insulin am Morgen.

Bei Verwendung von lang wirksamem Basalinsulin: Gabe der gesamten Morgendosis (bei zweizeitiger Gabe) bzw. der gesamten Tagesdosis (bei einzeitiger Gabe).

Am Morgen der Operation Glukose 5 % mit 75–125 ml/h (entsprechend 3,75–6,25 g Glukose/h), um kataboler Stoffwechsellage entgegenzuwirken.

Blutglukose stündlich messen, häufiger bei Blutglukose < 100 mg/dl oder bei raschem Absinken der Blutglukose. Bei kritisch kranken Patient:innen arterielle/venöse Bestimmung der Blutglukose.

### Lange und komplexe Eingriffe

In der Regel ist eine intravenöse Insulingabe notwendig.

Kontrollen der Blutglukose in < 1-stündlichem Abstand (häufiger bei Blutglukose < 100 mg/dl oder bei raschem Absinken der Blutglukose).

Engmaschige Elektrolytkontrollen für die Dauer der i.v.-Insulintherapie.

Beginn: früh am Morgen vor der Operation.

#### Cave

Eine Insulingabe ist bei Menschen mit Typ-1-Diabetes durchgehend erforderlich, um einer Ketoazidose entgegenzuwirken. Der basale Insulinbedarf ist immer sicherzustellen. Eine alleinige Korrekturinsulingabe ist inadäquat.

## Orale Antidiabetika/Therapie mit GLP-1-Rezeptoragonisten im Krankenhaus

In den meisten Fällen erfolgt eine Spitalaufnahme nicht zur Adaption einer oralen antidiabetischen Therapie/Therapie mit GLP-1-Rezeptoragonisten. Daher sollte man prinzipiell bei einer Krankenhausaufnahme aufgrund der zur Aufnahme führenden Krankheit immer eine passagere Insulintherapie andenken, da diese besser steuerbar ist als die antidiabetische Heimtherapie [20]. Bei Umstellung von einer Insulintherapie auf die antidiabetische Heimtherapie sollte allerdings die Heimtherapie 1 bis 2 Tage vor der geplanten Entlassung wieder initiiert werden, um eine evtl. Minderversorgung rasch zu erkennen. Bei kurzen Krankenhausaufenthalten, fehlenden Kontraindikationen und keiner akuten Stoffwechselentgleisung kann die antidiabetische Heimtherapie auch beibehalten werden [44, 45].

Die Initiierung einer oralen antidiabetischen Therapie/Therapie mit GLP-1-Rezeptoragonisten zur Behandlung vor allem einer Stresshyperglykämie bzw. akuter Erkrankung wird bei hospitalisierten Patient:innen nicht empfohlen [20, 46]. Je klinischem Verlauf kann dann eine derartige Therapie eingeleitet werden.

Bei Patient:innen mit bekanntem Diabetes mellitus soll zum Zeitpunkt der Krankenhausaufnahme der HbA_1c_-Wert bestimmt werden.

Prinzipiell können alle oralen Antidiabetika auch beim hospitalisierten, nicht kritisch kranken Patient:innen weiterverwendet werden. Hervorzuheben ist jedoch die Bedeutung der Vermeidung von Hypoglykämien bei Beibehaltung der Heimtherapie im Krankenhaus sowie die häufig akut einsetzenden Beeinträchtigungen der Eliminationsorgane Leber und Niere.

Jedoch hat jede Substanzklasse bestimmte Einschränkungen, die folgend aufgezählt werden.

## Metformin

### Metformin & Kontrastmittelgabe

#### Iod-basierte Kontrastmittel

Bei iod-basierten Kontrastmitteln ist folgende Vorgehensweise empfohlen [47, 48]:

1. Patient:innen mit eGFR > 60 ml/min/1,73 m^2^ können Metformin normal weiternehmen.
2. Patient:innen mit eGFR 30–59 ml/min/1,73 m^2^:
  i. Bei intravenöser Kontrastmittelgabe bei einer eGFR ≥ 45 ml/min/1,73 m^2^ kann Metformin weitergegeben werden.
  ii. Bei intraarterieller oder intravenöser Kontrastmittelgabe und einer eGFR von 30–44 ml/min/1,73 m^2^ sollte Metformin 48 h vor Kontrastmittelgabe pausiert werden und erst wieder begonnen werden, wenn sich die Nierenfunktion nachweislich nicht verschlechtert hat.
3. Metformin ist im Notfall keine Kontraindikation für notwendige Untersuchungen. Um das Risiko einer Laktatazidose zu reduzieren, sollte nach der Untersuchung Metformin jedenfalls 48 h pausiert werden.

#### Gadolinium-basierte Kontrastmittel

Bei Gadolinium-basierten Kontrastmitteln sind keine speziellen Vorkehrungen notwendig [47].

### Metformin & Niereninsuffizienz

Metformin darf bei eingeschränkter Nierenfunktion bis zu einer eGFR von 30 ml/min/1,73 m^2^ bei fehlenden anderen Risikofaktoren für eine Laktatazidose eingesetzt werden [49]. Allerdings sollte es bei Patient:innen mit einer eGFR von 30–45 ml/min/1,73 m^2^ in reduzierter Dosis mit maximal 1000 mg täglich verwendet werden.

### Metformin-assoziierte Laktatazidose

Die Inzidenz der Metformin-assoziierte Laktatazidose (MALA; [48, 50]) wird mit 3 bis 10/100.000 Patient:innenjahre angegeben.

Die Ursache einer MALA ist bis heute nicht restlos geklärt. Am Beginn dürfte ein plötzlicher rascher Anstieg der Metforminkonzentration im Blut stehen, welcher bei eingeschränkter Leberfunktion eine Laktatproduktion triggern kann. Zur Akkumulation von Laktat und Metformin, welche dann zu einer Laktatazidose führen, kommt es beim Vorliegen bestimmter Begleitumstände wie einem akuten Nierenversagen, Sepsis, Herz-Kreislauf-Versagen, Alkoholismus, Leberzirrhose und anderen hypoxischen Zuständen (z. B. Schock).

An eine MALA sollte bei unspezifischen abdominellen Beschwerden in Verbindung mit Muskelkrämpfen gedacht werden. Eine Blutgasanalyse bestätigt das Ergebnis bei vermindertem pH-Wert und erhöhten Laktatspiegeln (> 5,0 mmol/l) [51].

Die Bestimmung von Serumkonzentrationen von Metformin wird empfohlen, da dadurch die Verdachtsdiagnose bestätigt werden kann, hat aber keinen Einfluss auf die Therapie. Neben dem Absetzen von Metformin steht die Behandlung der Grundkrankheit im Vordergrund. Da Metformin nicht an Albumin gebunden ist, kann es durch eine Hämodialyse eliminiert werden. Allerdings hängt die Prognose der Patient:innen nicht von der Höhe der Metforminkonzentration ab, sodass die Indikation zur Hämodialyse eher aufgrund eines evtl. auch bestehenden Nierenversagens gestellt wird.

### Metformin & Erkrankungen

Bei Patient:innen, die aufgrund von schweren Infektionen, dekompensierter oder instabiler Herzinsuffizienz, Leberversagen oder auch schwerer Diarrhö und Exsikkose hospitalisiert wurden, muss Metformin pausiert werden.

## Pioglitazon

Pioglitazon darf nicht bei Patient:innen mit Herzinsuffizienz (durch erhöhte Natriumrückresorption kommt es zu einer Flüssigkeitsretention) und bei eingeschränkter Leberfunktion (ALT > 2,5 × der Obergrenze des Normbereichs) angewandt werden.

## Sulfonylharnstoffe und Glinide

Aufgrund ihres Wirkmechanismus kann es unter Sulfonylharnstofftherapie zu Hypoglykämien kommen. Bei eingeschränkter Nierenfunktion kann die Akkumulation v. a. der lang wirksamen Sulfonylharnstoffe zu Hypoglykämien führen. Diese sollten daher bei hospitalisierten Patient:innen mit Vorsicht angewandt werden. Eine Studie zeigte mehr Hypoglykämien bei hospitalisierten Patient:innen unter Sulfonylharnstoffen verglichen zu Kontrollen [52]. Aber auch bei Patient:innen, die aufgrund einer interkurrenten Erkrankung im Krankenhaus weniger Nahrung zu sich nehmen, sollte die Sulfonylharnstofftherapie reduziert bzw. pausiert werden.

Bei Vorliegen einer Hyperglykämie unter Sulfonylharnstofftherapie beim hospitalisierten Patient:innen ist die Umstellung auf eine zumindest passagere Insulintherapie indiziert.

Bei eingeschränkter Leberfunktion kann durch die gestörte hepatische Glukoneogenese das Hypoglykämierisiko deutlich erhöht sein. Glinide werden vorwiegend hepatisch eliminiert und sind daher bei Patient:innen mit Leberversagen kontraindiziert.

## Dipeptidyl-Peptidase-IV-Inhibitoren

Dipeptidyl-Peptidase-IV (DPP-IV)-Inhibitoren haben ein sehr geringes Hypoglykämierisiko und können daher auch bei Patient:innen mit eingeschränkter Nierenfunktion in adaptierter Dosis bei hospitalisierten Patient:innen verwendet werden. Erste Studiendaten zeigen auch, dass DPP-IV-Inhibitoren beim hospitalisierten Patient:innen gemeinsam mit einem Basalinsulin eine gleich gute Blutglukosesenkung erzielten wie Patient:innen mit einem lang und kurz wirksamen Insulin [53, 54]. Für Alogliptin und Saxagliptin besteht eine Warnung der FDA bezüglich Herzinsuffizienz [55]. Beide Substanzen sollten daher bei hospitalisierten Patient:innen mit Herzinsuffizienz nicht eingesetzt werden. Außerdem sollte bei Patient:innen mit nicht biliärer Pankreatitis in der Anamnese oder chronischer Pankreatitis eine DPP-IV-Inhibitortherapie nicht eingeleitet werden bzw. die Therapie bei Pankreatitisanamnese langfristig abgesetzt werden.

## GLP-1-Rezeptoragonisten

GLP-1-Rezeptoragonisten (GLP-1-RA) haben ein sehr geringes intrinsisches Hypoglykämierisiko. Aufgrund positiver Endpunktdaten für Liraglutide, Albiglutide, Semaglutide und Dulaglutide (LEADER-Studie, HARMONY-Studie, SUSTAIN-6-Studie, REWIND-Studie) sollte eine bestehende GLP-1-RA-Therapie gerade bei kardiovaskulär kranken Patient:innen aber auch bei Patient:innen mit chronischer Niereninsuffizienz nicht beendet werden [56–59].

Ausgenommen davon sind Aufnahmen aufgrund gastrointestinaler Ursachen (z. B. Übelkeit, Erbrechen etc.). Es ist zu berücksichtigen, dass GLP-1-RA zu einer verzögerten Magenentleerung und folglich zu gastrointestinalen Beschwerden führen können.

## SGLT2-Inhibitoren

SGLT2-Inhibitoren sind klassische „sick day off“ Medikamente und sollten daher in den meisten Fällen bei hospitalisierten Patient:innen pausiert werden. Dies gilt insbesondere vor Operationen, bei längeren Fastenperioden oder auch bei interkurrenten, schwerwiegenden Erkrankungen, wenn Ketonkörper vorhanden sind. [60, 61].

Generell haben SGLT2-Inhibitoren ein geringes Hypoglykämierisiko, und das auch nur, wenn sie in Kombination mit Insulin und/oder Sulfonylharnstoffen/Gliniden eingesetzt werden. Dies muss bei hospitalisierten Patient:innen bedacht werden. Eine seltene, aber potenziell gefährliche Nebenwirkung unter dieser Therapie ist die euglykämische Ketoazidose, die v. a. bei plötzlich erhöhtem Insulinbedarf oder akuter Nierenfunktionsverschlechterung auftreten kann. Durch eine venöse Blutgasanalyse kann dies sehr einfach diagnostiziert werden, diese sollte bei allen Patient:innen mit klinischen Symptomen einer Ketoazidose und einem SGLT2-Inhibitor in der Medikation durchgeführt werden.

## Steroidtherapie

Steroide können sowohl über eine Zunahme der Insulinresistenz als auch einer Betazellfunktionsstörung zu einer Hyperglykämie führen [62].

Epidemiologische Studien zeigen, dass im Krankenhaussetting bis zu 86 % jener, die orale oder intravenöse Glukokortikoide erhalten, zumindest eine hyperglykämische Episode aufweisen [63]. Als Risikofaktoren für das Auftreten einer Hyperglykämie wurden ein Alter > 65 Jahre, ein erhöhter BMI, eine positive Familienanamnese für Diabetes mellitus, ein HbA_1c_ ≧ 6,0 % (42 mmol/mol) vor der Steroidtherapie oder auch eine hohe Steroiddosis identifiziert [64]. In verschiedenen Patient:innenpopulationen konnte eine Assoziation zwischen dem Auftreten einer steroidassoziierten Hyperglykämie und dem Outcome der Patient:innen gezeigt werden [65, 66].

Nachdem kurz wirksame Glukokortikoide wie Prednisolon eine Wirkspitze nach 4–8 h aufweisen, bietet sich eine Therapie mit einem NPH-Insulin an (Tab. 2 zur Dosisempfehlung [67]). Für länger wirksame Steroide wie Dexamethason oder bei mehrmalig täglichen Gaben bieten sich länger wirksame Basalinsuline (Insulin degludec, Insulin glargin, Insulin glargin U300) an. Bei höheren Glukokortikoiddosen können zusätzliche prandiale Insulinapplikationen notwendig sein. Liegt bereits ein Diabetes mellitus mit Insulintherapie vor der Steroidtherapie vor, so sollte die Insulindosis unter Steroiden um zumindest 20 % angehoben werden. Weitere Insulindosisanpassungen unter regelmäßigen Blutglukosekontrollen sind essenziell.

**Table 2:** 

Prednisolonäquivalentdosis (mg)	Insulindosis (IE/kgKG)
≥ 40	0,4
30	0,3
20	0,3
10	0,1

## Anwendung von Diabetestechnologie im Krankenhaus

Insulinpumpen und kontinuierliche Glukosemonitoringsysteme finden eine zunehmend breitere Anwendung bei Patient:innen insbesondere mit Typ-1-Diabetes, in selteneren Fällen werden sie auch von Patient:innen mit Typ-2-Diabetes angewandt. Eine Verwendung derartiger Technologien setzt voraus, dass die Anwender selbst mit der Bedienung vertraut sind und allfällig auftretende Probleme eigenständig zu lösen wissen. Randomisierte kontrollierte Studien, welche untersuchen, ob es dadurch einen klinischen Benefit gibt, sind rar und nur auf ein einzelnes CGM-System beschränkt. Details zur Insulinpumpentherapie und kontinuierlichen Glukosemessung sind in den jeweiligen Kapiteln dargestellt, im Folgenden wird nur die Sondersituation des stationären Aufenthaltes behandelt.

## Insulinpumpentherapie im Krankenhaus

Durch die steigende Anzahl an Insulinpumpenträgern nimmt auch die Zahl an hospitalisierten Patient:innen mit Pumpentherapie zu, deshalb ist es notwendig sich mit dieser Patient:innenpopulation genauer auseinanderzusetzen. Patient:innen, welche körperlich und geistig in der Lage sind, ihre Insulinpumpentherapie selbstständig fortzusetzen, können dies auch während des stationären Aufenthalts tun. Allerdings ist es notwendig, dass die betreffenden Krankenhäuser definierte Regeln für deren Anwendung haben, dass auf die Selektion geeigneter Patient:innen geachtet wird und dass aktive Kommunikation zwischen Patient:innen und medizinischem Personal bezüglich der Insulinpumpentherapie erfolgt, damit eine sichere Anwendung ohne Patient:innengefährdung gegeben ist [68–71]. Des weiteren sollte nach Möglichkeit das Personal Erfahrung im Umgang mit Insulinpumpen haben [72]. Generell haben jedoch die Patient:innen selbst meist mehr Erfahrung im Umgang mit Insulinpumpen als das betreuende Team auf der Bettenstation. Der Fokus sollte in dem Fall auf Pumpenselbstmanagement gelegt werden, welches auch zu einer größeren Patient:innenzufriedenheit führt [73, 74]. Eine retrospektive Analyse zeigte eine geringere Anzahl an schweren Hypoglykämien (< 40 mg/dl) und Hyperglykämien (> 350 mg/dl) bei Fortsetzung der Insulinpumpentherapie bei etwa gleicher Glukoseeinstellung [72]. Kannan et al. konnten zeigen, dass unter Fortsetzung von Insulinpumpentherapie eine vergleichbare Glukoseeinstellung ohne vermehrte Hypo- oder Hyperglykämien erreicht werden kann, wenn sie in geeigneten Situationen und bei geeigneten Patient:innen angewandt wird [75].

**Figure 2:**
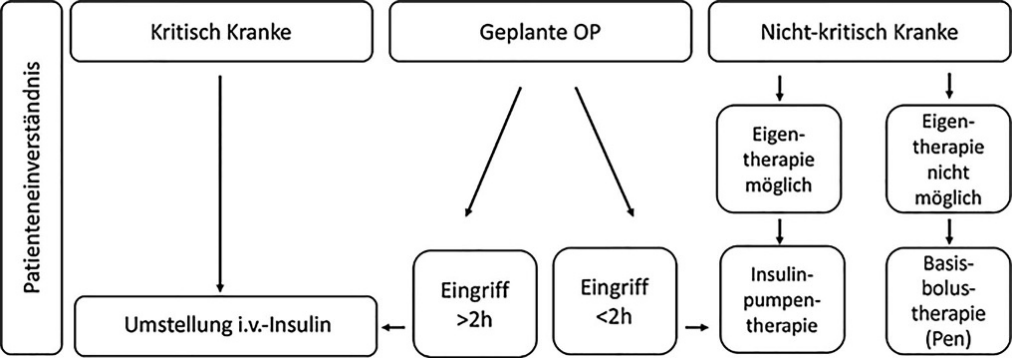

Die Abb. 2 stellt dar, unter welchen medizinischen Bedingungen eine Insulinpumpentherapie stationär fortgesetzt werden kann bzw. wann sie (zwischenzeitlich) beendet werden sollte.

Wenn die Insulinpumpentherapie unter stationären Bedingungen fortgesetzt wird, sollte auch das Diabetesteam des betreffenden Krankenhauses im Verlauf des Aufenthaltes hinzugezogen werden, um die Therapie zu evaluieren. Des Weiteren sollten Pumpentyp, Insulintyp und Pumpeneinstellungen dokumentiert werden. Therapieempfehlungen bzw. Dosisänderungen (Basalrate, Bolusdosis, Korrekturfaktor, Blutglukosemessfrequenz, Glukosezielbereich) müssen dokumentiert werden, und die Umsetzung durch die Patient:innen sollte in regelmäßigen Abständen evaluiert werden. Es muss in zeitnahen Abständen überprüft werden, ob die Patient:innen weiterhin in der Lage sind, die Therapie selbst zu steuern [20, 76]. Patient:innen sollten keine Änderung der Pumpeneinstellungen vornehmen, ohne diese mit dem betreuenden Team zu besprechen. Des Weiteren wird empfohlen die Patient:innen auf die Zielbereiche im Krankenhaus hinzuweisen und wenn notwendig diese in den Pumpeneinstellungen für den Zeitraum des stationären Aufenthalts anzupassen [77].

Während des stationären Aufenthalts ist sicherzustellen, dass ausreichend Bedarfsmaterial für die Insulinpumpentherapie vorhanden ist. Katheterwechsel haben in gewohnter Regelmäßigkeit zu erfolgen. Durch das nosokomiale Keimspektrum ist besonderes auf Infektionen im Bereich der Setzstellen zu achten [78].

Die Tab. 3 fasst die Kontraindikationen zur Fortsetzung der Insulinpumpentherapie im Krankenhaus zusammen.

**Table 3:** 

Veränderter Bewusstseinszustand (außer bei kurzer Anästhesie)
Patient*in zeigt sich nicht in der Lage, die Pumpe adäquat zu bedienen
Intensivpflichtigkeit
Psychiatrische Erkrankung (z. B. schwere Depression und/oder Suizidalität), die ein Diabetes-Selbstmanagement unmöglich macht
Diabetische Ketoazidose oder hyperosmolarer hyperglykämischer Zustand
Unwillen der Patient*innen, die Insulinpumpentherapie fortzusetzen
Mangel an Insulinpumpenzubehör
Mangel an qualifiziertem Fachpersonal (Diabetolog*innen, Diabetesberater*innen)
Entscheidung aus medizinischen Gründen

Sollte eine Fortsetzung der Insulinpumpentherapie nicht möglich sein, muss je nach Gesundheitszustand eine Umstellung auf eine subkutane Basis-Bolus-Therapie erfolgen oder im Fall einer akuten Verschlechterung (Intensivpflichtigkeit, größere Operation, Ketoazidose) auf eine intravenöse Insulintherapie umgestellt werden. Die Basalrate muss bei subkutaner Insulintherapie entsprechend durch ein Basalinsulin ersetzt werden. Die Bolusinsulindosis kann entweder mit 1/6 der bisherigen Insulintagesdosis zu den 3 Hauptmahlzeiten angenommen werden oder errechnet nach Insulin/Broteinheiten-Verhältnis verabreicht werden. Ein Korrekturschema sollte zudem vorgegeben sein [79].

Während des stationären Aufenthaltes kommt es immer wieder zu invasiven und nichtinvasiven Untersuchungen oder Operationen. Welche Maßnahmen hinsichtlich einer etablierten Insulinpumpentherapie zu treffen sind, fasst Tab. 4 nach Mendez zusammen [40].

**Table 4:** 

Röntgen/CT	Pumpe soll mit einer Bleischürze geschützt werden
MRT	Pumpe und Infusionsset aus Stahl müssen entfernt werden
Ultraschall	Pumpe kann an Ort und Stelle bleiben, der Schallkopf soll nicht direkt auf die Pumpe zusteuern
Herzkatheteruntersuchung	Pumpe soll mit einer Bleischürze geschützt werden
Schrittmacher‑/Defibrillator-Implantation	Pumpe soll mit einer Bleischürze geschützt werden
Koloskopie/Gastroskopie	Pumpe kann an Ort und Stelle bleiben
Laserchirurgie	Pumpe kann an Ort und Stelle bleiben

*CT* Computertomographie, *MRT* Magnetresonanztomographie

Vor der Entlassung sollte eine Konsultation des behandelnden Diabetesteams erfolgten, sodass Pumpeneinstellungen überprüft und an die geänderten Bedingungen nach dem Krankenhausaufenthalt angepasst werden können [79].

## Kontinuierliche Glukosemessung im Krankenhaus

Die kontinuierliche Glukosemessung (CGM) bietet den Vorteil, dass sie im Gegensatz zu kapillären Blutglukosemessungen ein kontinuierliches Signal mit Trends sichtbar macht und – abhängig vom System – auch vor Blutzuckerentgleisungen warnt. Aktuell ist keines der kommerziellen Systeme, welche die Glukose im subkutanen Gewebe messen, für die Anwendung im Krankenhaus zugelassen. Dennoch kann es durchaus sein, dass Patient:innen ihr CGM-System weiterverwenden möchten, um die Therapie bestmöglich fortzusetzen. Dem spricht nichts entgegen, allerdings ist es forensisch sicherlich von Vorteil, an definierten Zeitpunkten ergänzend kapilläre Messungen über das hauseigene Point of Care (POC)-System vorzunehmen.

Neben den generell bekannten Faktoren (Zeitverzögerung des subkutanen Signals, Sensordrift, Notwendigkeit der regelmäßigen Kalibration, Kalibration unter stabiler Glykämie), welche die CGM-Genauigkeit beeinflussen, kann die Genauigkeit des subkutanen CGM-Signals insbesondere unter stationären Bedingungen durch bestimmte Situationen (Harnsäurekonzentration, Dehydratation, Vasokonstriktion, Hypotonie, Hypothermie, Hypoxie, stark fallende Blutzuckerkonzentration) sowie Medikamente (Paracetamol, Maltose, Ascorbinsäure, Mannitol, Heparin, Salicylsäure, Hydroxyurea) zusätzlich beeinflusst werden [80–82].

Studien zeigen allerdings eine gute Übereinstimmung von Werten, die mittels kapillärer POC-Messung und CGM gemessen wurden [83]. Insbesondere die kritische Phase der Nacht könnte durch CGM besser dargestellt werden und möglicherweise sonst nicht erfasste Hypoglykämien darstellen [84]. Auch für isCGM konnten vergleichbare Ergebnisse gezeigt werden [85]. Aktuell liegen 2 randomisiert kontrollierte Studien zum CGM Einsatz bei hospitalisierten Patient:innen vor. Diese zeigten eine Verbesserung der glykämischen Kontrolle anhand der Zeit im Zielbereich, sowie eine Reduktion von Hypoglykämien [86].

Nachdem die CGM-Systeme (insbesondere Patient:innengeräte, welche von zu Hause mitgebracht wurden) zumeist nicht an das hausinterne Telemetriesystem angeschlossen sind, werden Pflegekräfte vor kritischen Blutzuckerentgleisungen nicht gewarnt, wenn der Alarm nur lokal im Patient:innenzimmer anschlägt. Dies könnte ein rechtliches Problem sein. Telemetrie per CGM könnte möglicherweise in Zukunft die stationäre Behandlung von Patient:innen mit Diabetes verbessern. In einer Pilotstudie zeigte der Einsatz eines CGM-Telemetriesystems nicht nur eine Verbesserung der glykämischen Kontrolle, sondern auch Vorteile in Bezug auf Patient:innenschulung [87]. In einer randomisiert kontrollierten Studie konnten Singh et al. zeigen, dass ein CGM-Telemetriesystem bei Patient:innen mit erhöhtem Hypoglykämierisiko die Rate an Hypoglykämien (< 70 mg/dl) verglichen mit POC-Messungen reduzieren kann [88].

Hinsichtlich Entfernung von Sensoren vor bildgebenden Untersuchungen ist hier dasselbe Vorgehen wie bei der Insulinpumpentherapie indiziert (Tab. 4).

## Hybrid Closed Loop im Krankenhaus

Hybrid Closed Loop Systeme (bestehend aus Insulinpumpe, CGM-System und Algorithmus zur Steuerung der Insulinabgabe) kommen als Weiterentwicklung der Sensor-unterstützten Pumpentherapie in den letzten Jahren vermehrt zum Einsatz. Die derzeit verfügbaren Modelle sind die Medtronic 670G/780G und die Ypsopump mit dem CamAPS FX. Bei Patient:innen, die ein derartiges System zum Diabetesmanagement verwenden, werden bei Aufnahme in den stationären Bereich dieselben Anforderungen wie an Insulinpumpenträger oder CGM-Nutzer:innen gestellt. Auch hier wird darauf hingewiesen, dass nur Patient:innen mit ausreichendem Wissen und Fähigkeiten im Umgang mit ihrem Hybrid Closed Loop System ihre Therapie während des stationären Aufenthalts fortführen sollten und diese Entscheidung regelmäßig überprüft werden soll. Des Weiteren sollte auch hier ein definiertes Prozedere im Umgang mit derartigen Systemen festgelegt werden [89].

Für die Anwendung von Hybrid Closed Loop Systemen (Artificial Pancreas bzw. automatisierten Insulindosierungssystemen) im stationären Bereich stehen bisher keine kommerziellen Systeme explizit dafür zur Verfügung. Jedoch wurden derartige Systeme bereits in der stationären Versorgung untersucht. Die Anwendung eines Hybrid Closed Loop Systems bei Patient:innen mit Typ-2-Diabetes auf der Normalstation zeigte deutlich bessere Glukoseeinstellung ohne erhöhtes Hypoglykämierisiko, höhere Zeit im Zielbereich und reduzierte Glukosevariabilität verglichen mit subkutaner Insulintherapie [90, 91]. Ebenso zeigte sich eine hohe Patient:innenzufriedenheit unter der Therapie.

## Entscheidungsunterstützungssysteme für Diabetesmanagement im Krankenhaus

Bisher erfolgt in den meisten Fällen die Dokumentation von Blutglukosewerten manuell auf den sogenannten „Diabeteskurven“. Auf diesen wird auch die jeweilige Insulindosis dokumentiert. Oft sind derartige Dokumente schwer leserlich geführt, es kann zu Übertragungsfehlern (Glukosewerte, Insulindosen) kommen, und trotz bestehender Guidelines werden Insulindosen aus Angst vor Hypoglykämien nur zögerlich gesteigert [92, 93]. Elektronische Diabetesmanagementsysteme mit integrierter Entscheidungsunterstützung können Blutglukosewerte direkt aus dem Laborinformationssystem importieren, grafisch darstellen und einen Insulindosisvorschlag für den jeweiligen Zeitpunkt geben [94]. Durch derartige Systeme wird das Diabetesmanagement besser visualisiert und es kommt zu einer geringeren Fehlerhäufigkeit. Die Anwendung der Insulindosisvorschläge führt zu einer besseren Blutglukoseeinstellung, auch während intrahospitaler Fastenphasen [95, 96]. Aktuell gibt es in Europa ein CE-zertifiziertes System (GlucoTab, decide Clinical Software GmbH), in den USA ist ebenfalls ein System (Glucommander, Glytec) von der Food and Drug Administration (FDA) zugelassen.

## Interessenkonflikt

J.K. Mader: Vortragshonorare von AstraZeneca, Abbott Diabetes Care, Böhringer-Ingelheim, Eli Lilly, Medtronic Minimed, Menarini, Merck Sharp & Dohme, Nintamed, Novo Nordisk, Roche Diabetes Care, Sanofi-Aventis, Servier, Viatris, Wellion und Ypsomed. Advisory Board: Abbott Diabetes Care, Böhringer-Ingelheim, Eli Lilly, Medtronic Minimed, Novo Nordisk, Pharmasense, Roche Diabetes Care, Sanofi-Aventis. Gesellschafterin decide Clinical Software GmbH. Forschungsgelder von A. Menarini Diagnostics, B. Braun, Metronom Health, Novo Nordisk, Profusa Inc., Roche Diabetes Care, Sanofi-Aventis. J. Brix: Honorare von AstraZeneca, Bayer, Boehringer-Ingelheim, Eli Lilly, MSD, Novartis, Novo Nordisk. J.M. Brix: Vortragshonorare oder Advisory Boards: Abbott, Boehringer-Ingelheim, Eli Lilly, MSD, NovoNordisk, sanofi-aventis. F. Aberer: Vortragshonorare von AMGEN, Astra Zeneca, NovoNordisk, Boehringer Ingelheim, Eli Lilly, Sanofi Aventis und Forschungsgelder von Sanofi Aventis. A. Vonbank: Vortrags- und Beratungshonorare Boehringer-Ingelheim, Astra Zeneca, Sanofi-Aventis, Amgen, Novo Nordisk, Eli Lilly, Roche, Daichii Sankyo. M. Resl: Vortragshonorare von Novo Nordisk, Viatris, Dexcom, Boehringer-Ingelheim. D.A. Hochfellner: Reisehonorare von Eli Lilly, Novo Nordisk, Novartis und MSD. C. Ress: Vortragshonorare und Kongressunterstützung von AstraZeneca, Novo Nordisk, Pfizer, Boehringer Ingelheim, Amryt und Sanofi. T.R. Pieber: Direktor des Institutes für Biomedizin und Gesundheitswissenschaften (HEALTH) von JOANNEUM RESEARCH. Wissenschaftlicher Vorstand von CBmed (Zentrum für Biomarkerforschung). Vortragshonorare von der Firma Novo Nordisk. Mitglied von Advisory Boards von den Firmen Arecor, Novo Nordisk, Sanofi, AstraZeneca, Adocia. L. Stechemesser: Forschungsunterstützungen und/oder Honorare von Abbott, AstraZeneca, Boehringer-Ingelheim, Eli Lilly, Medtronic, MSD, Novartis, Novo Nordisk, Roche, Sanofi-Aventis. H. Sourij Vortragshonorare oder Advisory Boards: Amgen, AstraZeneca, Bayer, Boehringer Ingelheim, Daiichi Sankyo, Eli Lilly, Kapsch, Merck Sharp & Dohme, Novartis, Novo Nordisk, Roche Diagnostics, Sanofi-Aventis. Forschungsuntersgrants: Boehringer Ingelheim, Eli Lilly, Merck Sharp & Dohme, Novo Nordisk, Sanofi-Aventis.
